# A case of systemic sclerosis/lupus overlap syndrome presenting with bilateral cotton wool spots

**DOI:** 10.1016/j.ajoc.2020.100967

**Published:** 2020-10-10

**Authors:** Caleb Liles, Chase Warner, Ronald Warwar

**Affiliations:** a Ohio University Heritage College of Osteopathic Medicine, Athens, OH, United States; b Department of Ophthalmology, Grandview Medical Center, Dayton, OH, United States; c Wright State University, Dayton, OH, United States

**Keywords:** Cotton wool spots, Systemic lupus erythematosus, Systemic sclerosis, Overlap syndrome

## Abstract

**Purpose:**

Systemic sclerosis (SSc) is characterized by multi-system inflammation and fibrosis. Ophthalmologists must be aware of the uncommon ocular features of SSc to secure the diagnosis.

**Observations:**

Here we report the rare occurrence of bilateral cotton wool spots in an 86-year-old woman with SSc/lupus overlap syndrome presenting with a history of chronic obstructive pulmonary disease, gastroesophageal reflux disease, polymyalgia rheumatica, scalp tenderness, and right jaw pain on chewing. Fundoscopy showed diffuse bilateral cotton wool spots that prompted the diagnosis of SSc/lupus overlap syndrome.

**Conclusions:**

The confluence of patient symptoms was disguised as separate diseases, but the funduscopic finding of cotton wool spots in a patient without known risk factors prompted further investigation and the correct diagnosis. She was started on immunosuppressant therapy but unfortunately died four months later after developing right heart failure.

**Importance:**

The differential diagnosis for bilateral cotton wool spots should include autoimmune processes such as SSc and systemic lupus erythematosus and may represent an early sign that can help direct treatment.

## 1 Introduction

Systemic sclerosis (SSc) is a diffuse autoimmune process affecting the microvasculature of the gastrointestinal (GI) tract, lungs, kidneys, heart, skin, and joints, with subsequent fibrosis of the involved organs. This disease can present alone or as part of an overlap syndrome with other autoimmune diseases including systemic lupus erythematosus (SLE), which occurs in approximately 7% of SSc cases.^1^^,^^2^ Early manifestations of SSc include Raynaud's phenomenon, gastroesophageal reflux disease (GERD), and musculoskeletal pain with constitutional symptoms. SSc is also associated with trigeminal or glossopharyngeal neuralgia, lung fibrosis with pulmonary hypertension, renal failure, GI complications, and microangiopathy.^3^ A rare sign of SSc is cotton wool spots (CWSs) on funduscopic examination signifying SSc-related microangiopathy.^4^ No disease-modifying treatments currently exist, but treatments targeted toward the affected organ system can improve function, and immunosuppressants can be used for pulmonary and cutaneous issues.

Here we present a case of SSc/lupus overlap syndrome that was finally diagnosed after detection of CWSs on fundoscopic examination. This case highlights the importance of considering autoimmune and collagen vascular diseases in the differential diagnosis when CWSs are observed by fundoscopy.

## 2 Case report

An 86-year-old woman with a history of chronic obstructive pulmonary disease (COPD) and GERD was referred to ophthalmology by rheumatology after being diagnosed with polymyalgia rheumatica (PMR). She reported scalp tenderness and right jaw pain on chewing without headaches or visual symptoms. She had a three-month history of worsening arthralgia and myalgia when diagnosed with PMR.

Her presumed COPD was controlled with oxygen and fluticasone/salmeterol, and her GERD was controlled with famotidine. There was no history of hypertension or diabetes, and her blood pressure was 94/68 with a heart rate of 100 on presentation. Her erythrocyte sedimentation rate (ESR) was 23 mm/h (normal 0–30 mm/h) and *C*-reactive protein (CRP) was elevated at 4.8 mg/L (normal <0.8 mg/L). She had been prescribed prednisone (10 mg b.i.d. for one week) by rheumatology. On examination, she was 20/40 OD 20/50 OS with no change from baseline. Pupils, motility, and anterior segment (PCIOL OU) were normal.

**Fig. 1:**
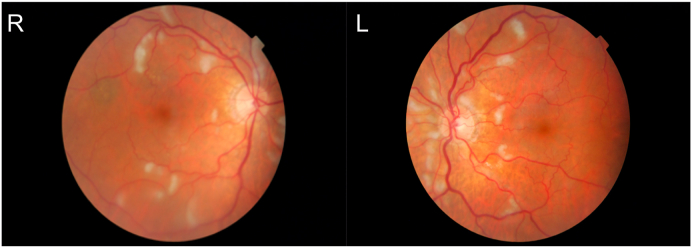
Fundus photos demonstrating diffuse bilateral cotton wool spots and vascular tortuosity.

**Fig. 2:**
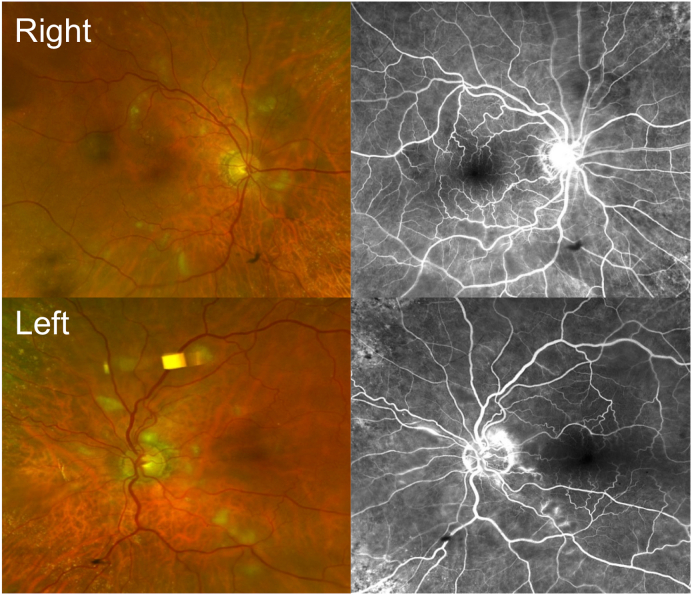
Early phase fluorescein angiogram showing hypofluorescence in the regions of cotton wool spots and hyperfluorescent regions of leaking indicative of vasculitis. Top row, right eye; bottom row, left eye.

Fundal examination revealed diffuse bilateral CWSs (Fig. 1). There were no other signs of retinopathy to suggest diabetes or hypertension such as macular edema, retinal exudates, flame or dot/blot hemorrhages, microaneurysms, arteriovenous nicking, or optic disk swelling. Fluorescein angiography showed regions of hypofluorescence in the regions of cotton wool spots and hyperfluorescent regions in the left eye indicative of vasculitis (Fig. 2). There was no evidence of retinal vascular occlusion or history consistent with malignancy, trauma, radiation exposure, or infection. Since the CRP was elevated on a background of presumed PMR, giant cell arteritis (GCA) remained the leading differential diagnosis, but other autoimmune processes were not ruled out. Subsequent temporal artery biopsy was normal, but with a 1.6 cm specimen (at least 2 cm is considered adequate).

**Fig. 3:**
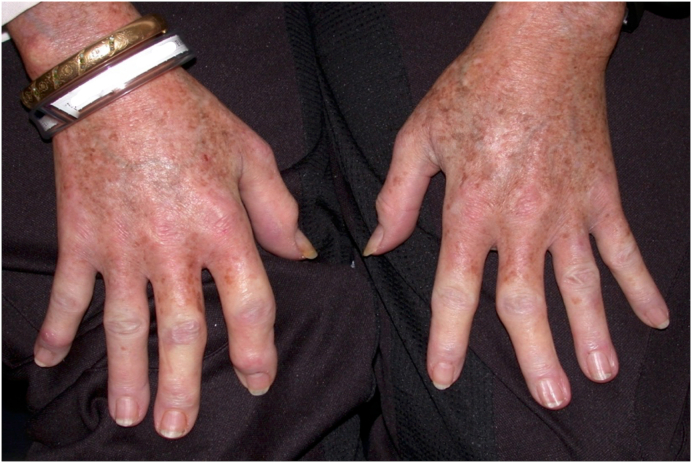
Patient's hands showing tight and thickened skin, and Raynaud's phenomenon.

The patient developed hypoxia, weakness, and swelling of the hands and was hospitalized. On further evaluation, she demonstrated Raynaud's phenomenon, tightening and thickening of the skin (Fig. 3), dysphagia, pulmonary hypertension, and pericardial and pulmonary effusions. Pulmonary hypertension was diagnosed by right heart catheterization. She was also found to have interstitial pulmonary fibrosis on computed tomography (CT) scan of the chest. Her renal function was normal, but anti-nuclear (ANA), anti-SSA (Ro), anti-double stranded DNA (anti-dsDNA) and anti-topoisomerase (anti-Scl-70) were positive. She was diagnosed with SSc/lupus overlap syndrome.

The patient was treated as an outpatient with mycophenolate mofetil. Unfortunately, four months later her pulmonary hypertension was complicated by right heart failure and she subsequently died.

## 3 Discussion/conclusions

Autoimmune conditions such as scleroderma and lupus can be challenging to diagnose due multisystem involvement and non-specific clinical findings. This case demonstrates the value of ophthalmologic examination in diagnosing systemic conditions including autoimmune diseases. CWSs were found on funduscopic examination without any other abnormalities, which prompted further consideration of the differential diagnosis. Though CWSs are common, an absence of risk factors or other signs suggestive of the more common etiologies such as diabetes or hypertension should prompt further workup and evaluation for rarer causes. To our knowledge, our case is the first of its kind to report CWSs in SSc/lupus overlap syndrome.

The patient's presentation highlights some of challenges in recognizing and diagnosing autoimmune disease. The arthralgia and myalgia experienced by this patient were likely to have been sclerodermal musculoskeletal pain, and the jaw claudication and scalp tenderness were likely sclerodermal trigeminal neuralgia, which is present in up to 4% of patients with SSc.^5^ Esophageal dysfunction is also commonly seen in SSc, with over 50% of patients affected and presenting with GERD, as seen here.^4^ The history also revealed COPD in this patient requiring oxygen and fluticasone/salmeterol, which masked the sclerodermal lung fibrosis, present in approximately 50% of patients with SSc.^6^ Though some of these findings might have been due to the lupus component, these complications are far more common in SSc.

The diagnosis of SSc can be made based on the 2013 American College of Rheumatology/European League Against Rheumatism Collaborative Initiative (ACR/EULAR) criteria.^7^ Skin thickening of the fingers, pulmonary arterial hypertension diagnosed by right heart catheterization, Raynaud's phenomenon and scleroderma related antibodies satisfy the criteria in our patient.

The diagnosis of SLE can be made based on the 2019 American College of Rheumatology/European League Against Rheumatism Collaborative Initiative (ACR/EULAR) criteria.^8^ In addition to a positive ANA as an “entry criterion”, anti-dsDNA, pericardial and pleural effusions satisfy the criteria in our patient.

Ocular manifestations of scleroderma include keratoconjunctivitis sicca, eyelid fibrosis, anterior uveitis, normal tension glaucoma, choroidal atrophy, and retinal ischemic vasculopathy.^4^ Though sclerodermal renal complications lead to hypertension in many patients, this patient was normotensive and renal function was normal. Minasian et al. reported CWSs in a normotensive scleroderma patient, though the additional findings included bilateral disc neovascularization and marked vascular tortuosity.^9^ Usiyama et al. studied retinal findings in normotensive SSc patients and found hard exudates and vascular tortuosities.^10^ Our case similarly demonstrates vascular tortuosity.

The lupus component of the overlap syndrome might have been responsible for the CWSs in this patient. Retinopathy occurs in 3–29% of SLE cases, depending on disease control, with CWSs being the most common retinal manifestation. Other findings that can be seen include venous or arterial occlusion, microaneurysms, arteriolar narrowing, retinal edema, or exudates.^11^ Though occlusive findings can be seen in Fig. 2, the other signs are not present. Greenan and Forester reported one SLE patient with active disease with CWSs who had adjacent capillary leakage on FA,^12^ which similarly was shown in our patient.

Therefore, the CWSs may have arisen from either the SSc or SLE component of the overlap syndrome in this case. If the CWSs were due to the lupus component, the clinical picture with predominant SSc features evolved to include subclinical features of increased disease activity of the lupus component. If the CWSs were due to the SSc component, this represents an ocular finding that has only rarely been reported in the literature,^13^ and even then secondary to SSc-related hypertension.^4^

Unfortunately, no disease-modifying drugs exist for SSc. Treatment is targeted toward the involved organ system such as interstitial lung disease, skin fibrosis, pulmonary hypertension, and renal failure. Immunosuppressants are used specifically to control pulmonary and cutaneous complications. In conclusion, CWSs must raise the suspicion of systemic autoimmune disease, especially when evidence of common causes of retinal ischemic vasculopathy is lacking.

## Patient consent

Consent to publish the case report was not obtained. This report does not contain any personal information that could lead to the identification of the patient.

## Funding

No funding was received for this work.

## Intellectual property

We confirm that we have given due consideration to the protection of intellectual property associated with this work and that there are no impediments to publication, including the timing of publication, with respect to intellectual property. In so doing we confirm that we have followed the regulations of our institutions concerning intellectual property.

## Research ethics

We further confirm that any aspect of the work covered in this manuscript that has involved human patients has been conducted with the ethical approval of all relevant bodies and that such approvals are acknowledged within the manuscript.

IRB approval was obtained (required for studies and series of 3 or more cases).

Written consent to publish potentially identifying information, such as details or the case and photographs, was obtained from the patient(s) or their legal guardian(s).

## Authorship

All listed authors meet the ICMJE criteria.  We attest that all authors contributed significantly to the creation of this manuscript, each having fulfilled criteria as established by the ICMJE.

We confirm that the manuscript has been read and approved by all named authors.

We confirm that the order of authors listed in the manuscript has been approved by all named authors.

## Declaration of competing interest

The authors have no conflicts of interest to declare.
